# Effect of Surgical Treatment for Deep Infiltrating Endometriosis on Pelvic Floor Disorders: A Systematic Review with Meta-analysis

**DOI:** 10.1055/s-0042-1742293

**Published:** 2022-02-17

**Authors:** Mirian Vieira Fraga, Cristina Laguna Benetti-Pinto, Daniela Angerame Yela, Ticiana Alves de Mira, Luiz Gustavo Oliveira Brito

**Affiliations:** 1 Department of Obstetrics and Gynecology, School of Medical Sciences, Universidade Estadual de Campinas, Campinas, SP, Brazil

**Keywords:** systematic review, endometriosis, fecal incontinence, urinary incontinence, pelvic floor, revisão sistemática, endometriose, incontinência fecal, incontinência urinária, assoalho pélvico

## Abstract

**Objectives** To evaluate the impact of surgical treatment of deep infiltrative endometriosis (DIE) on pelvic floor dysfunction (urinary incontinence [UI], pelvic organ prolapse [POP], fecal incontinence [FI)] or constipation, and sexual function [dyspareunia]).

**Data Source** The present systematic review was performed in the PubMed database. For the selection of studies, articles should be published by January 5, 2021, without language restriction.

**Study Selection** Six randomized controlled studies that evaluated surgical treatment for DIE and the comparison of different surgical techniques were included.

**Data Collection** The studies were selected independently by title and abstract by two authors. Disagreements were resolved by a third author. All included studies were also evaluated according to the Cochrane risk of bias tool and the quality of the evidence was analyzed using the GRADE criteria. Subgroup analysis by different treatments and follow-up periods was also performed.

**Results** Six studies were included in the quantitative analysis. The risk of bias between studies showed an uncertain risk of bias for most studies, with concealment of allocation being the least reported category. The quality of the evidence was considered low. High heterogeneity was found between the studies. No study has evaluated UI or POP comparatively before and after surgery.

**Conclusion** Dyspareunia and FI have improved after the surgical procedure, but it was not possible to demonstrate which surgical technique was related to these outcomes as there was surgical heterogeneity. This diversity was found across data, with the recommendation of future prospective studies addressing pelvic floor disorders with DIE.

## Resumo

**Objetivos** Avaliar o impacto do tratamento cirúrgico para endometriose infiltrante profunda (EIP) nas disfunções do assoalho pélvico (incontinência urinária [IU], prolapso de órgãos pélvicos [POP], incontinência fecal [IF] ou constipação e função sexual [dispareunia]).

**Fonte de Dados** A presente revisão sistemática foi realizada na base de dados PubMed. Para a seleção dos estudos, os artigos deveriam ser publicados até 5 de janeiro de 2021, sem restrição de idioma.

**Seleção dos Estudos** Foram incluídos seis estudos randomizados e controlados que avaliaram o tratamento cirúrgico para EIP e a comparação de diferentes técnicas cirúrgicas.

**Coleta de Dados** Os estudos foram selecionados de forma independente por título e resumo por dois autores. As discordâncias foram avaliadas por um terceiro autor. Todos os estudos incluídos foram avaliados de acordo com a ferramenta Cochrane de risco de viés e a qualidade de evidência foi analisada usando os critérios GRADE. A análise de subgrupo por diferentes tratamentos e períodos de acompanhamento também foi realizada.

**Resultados** Seis estudos foram incluídos na análise quantitativa. O risco de viés mostrou um risco incerto de viés para a maioria dos estudos, sendo a ocultação da alocação a categoria menos relatada. A qualidade de evidência foi considerada baixa. Alta heterogeneidade foi encontrada entre os estudos. Nenhum estudo avaliou a IU ou o POP comparativamente antes e após a cirurgia.

**Conclusão** A dispareunia e a IF melhoraram após o procedimento cirúrgico, mas não foi possível demonstrar qual técnica cirúrgica esteve relacionada a estes desfechos, pois houve heterogeneidade cirúrgica. Esta diversidade foi encontrada nos dados, com a recomendação de estudos prospectivos futuros abordando distúrbios do assoalho pélvico com EIP.

## Introduction

Endometriosis affects 10% of the female population. Its main symptoms are pelvic pain and infertility. For pain, women may refer dysmenorrhea, chronic pelvic pain, dyschezia, dysuria, and dyspareunia. 1 For deep infiltrative endometriosis (DIE), gastrointestinal manifestations (between 3.8 and 37%) 2 can be more intense and with major repercussions. The urinary tract can be involved (bladder endometriosis) in between 0.3 and 12% of the cases and may also compromise the quality of life of women. 3

The literature has several systematic reviews on the impact of conservative and/or surgical treatment of DIE. However, pelvic floor dysfunctions before and after treatment of endometriosis are not so deeply explored. When surgery is performed without focusing on nerve-sparing techniques or without carefully revising the anatomy, the risk for urinary incontinence (UI), fecal incontinence (FI), and other dysfunctions are possibly increased. An observational study assessing 138 women with DIE has shown that the presence of endometriosis in the bladder was an independent predictor of low bladder compliance, whereas the presence of endometriosis in the parametrium was predictor of voiding dysfunction. 4 5 6 A recent systematic review has found that colorectal surgery for endometriosis has a significant impact on urinary function regardless of the technique. 6 We can even find in the literature an association between bladder endometriosis and UI. 5

However, we do not have data pooled and analyzed into a systematic fashion, with analysis of the quality of evidence about pelvic floor dysfunctions and DIE or bladder endometriosis. Given that, we sought to systematically review the literature for studies that addressed pelvic floor dysfunctions with DIE before and/or after treatment.

## Methods

### Search Strategy

**Fig. 1:**
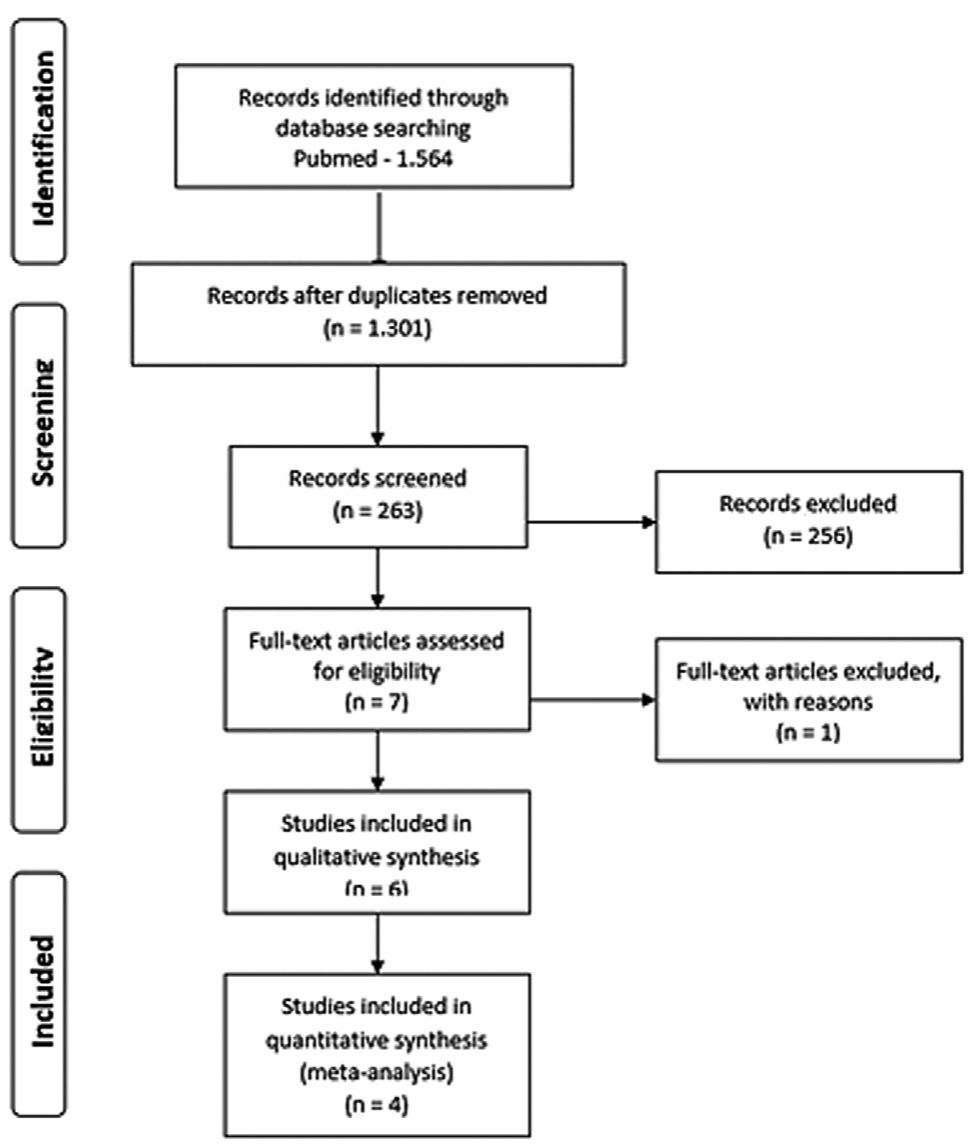
Flowchart diagram of identified studies.

The present systematic review was performed according to the preferred reporting items for systematic review and meta-analysis (PRISMA) guidelines 7 ( Fig. 1 ) and was registered in the PROSPERO database (CRD42020197049).

We have included randomized controlled studies that assessed surgical treatment for DIE and have compared the utilized techniques. We have excluded all studies that did not analyze pelvic floor dysfunctions, case reports, animal and/or experimental studies. The following outcomes were included: UI or FI, defined by self-report or any measurable, validated, or nonvalidated scale or questionnaire, following the IUGA/ICS recommendations for pelvic floor dysfunction terminology; pelvic organ prolapse (POP), whether symptomatic or by physical examination, constipation, and dyspareunia. Quality of life questionnaires related to pelvic floor dysfunctions were also included.

We have consulted the PubMed database on 5 January, 2021; no studies were excluded due to language restrictions. The following strategy was utilized: ((((((( *urinary incontinence* ) OR ( *incontinence* )) OR ( *fecal incontinence* )) OR ( *constipation* )) OR ( *pelvic organ prolapse* )) OR ( *prolapse* )) OR ( *urodynamics* )) OR ( *pelvic floor muscle* )) OR ( *dyspareunia* ) AND ( *endometriosis* ). We intended to produce a broad search strategy because we hypothesized that we would have difficulties to retrieve data.

### Study Selection, Data Extraction, and Risk of Bias

Studies were independently selected by title and abstract by two authors (Fraga M. V. and TAAM). Discordances were solved by a third author (Brito L. G. O.). Data extraction was performed in a previous spreadsheet pilot-tested and blinded for both authors. All included studies were also assessed according to the Cochrane risk of bias tool 8 9 and the quality of evidence was analyzed by the GRADE criteria. 9 Risk of bias analyzes five domains (selection, attrition, report, and other biases). The GRADE criteria consider the strength of other recommendations according to the presented variables.

### Data Analysis

Meta-analysis was considered when at least two studies could be pooled. Heterogeneity was classified according to the i 2 test 10 and a random-effect model was applied to data when i 2 was > 50%. Continuous variables were described as mean difference plus standard deviation (SD). Some outcomes were described as median plus interquartile ranges (IQRs) and their data were transformed into mean plus SD according to the following formula (median = mean, SD = IQR/1.35). Dichotomous variables were transformed into odds ratio (OR) plus 95% confidence intervals (CIs) with lower and upper limits. A subgroup analysis before and after treatment was performed for each treatment and pooled into forest plots. No funnel plots were built to assess publication bias as we did not have enough studies to perform this analysis. Statistical analysis was revised by Review Manager version 5.4 (The Cochrane Collaboration, Copenhagen).

## Results

### Study Selection and Characteristics

Fig. 1 depicts the process for data selection and extraction. After excluding duplicates, 1,301 studies were selected, and after another screening, 83 studies were fully read. Finally, 6 studies were selected, comprising 346 women. All manuscripts included women with DIE. 11 12 13 14 15 16

Four studies were performed in France, 11 12 13 14 one in Poland 15 and one in Italy. 16 For assessing dyspareunia, the visual analogic scale (VAS) 11 12 13 ; the numeric classification scale, 15 and the multidimensional punctuation system of Andersch 16 were used. For gastrointestinal symptoms, we have found the following questionnaires: Knowles – Eccersley – Scott (KESS) symptom questionnaire, gastrointestinal index of quality of life (GIQLI), and Wexner score. 12 13 14 For urinary symptoms, we have found the Urinary Symptom Profile (USP). 12 13 14 None of the studies criteria assessed UI and POP comparatively before and after surgery. Most studies analyzed dyspareunia. 11 12 13 15 16 Only three studies 12 13 14 have assessed the complaints of FI.

The surgical techniques compared were laparoscopically assisted or open colorectal resection surgery 11 ; conservative surgery (shaving or disc excision) or radical rectal surgery (segmental resection) 12 13 14 ; laparoscopy treatment using electroablation versus CO _2_ laser ablation, 15 and conservative surgery alone or conservative surgery and presacral neurectomy. 16

The primary outcomes of the studies were characterized by the relief of dyspareunia and the evaluation of gastrointestinal symptoms (constipation and fecal loss). The other outcomes proposed by the review were not analyzed as they were not found during data selection/extraction.

### Risk of Bias and GRADE Evaluation

**Fig. 2:**
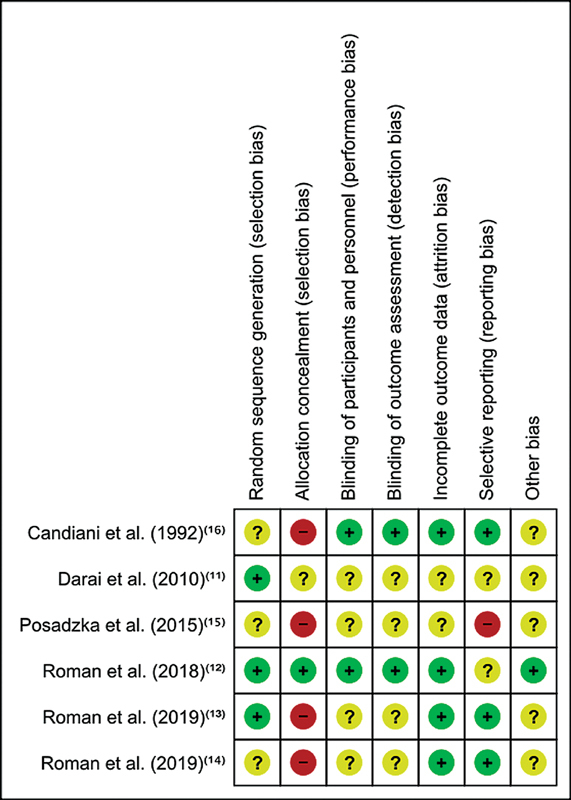
Risk of bias summary.

Three studies reported having performed a sample size calculation. 12 14 16 Two studies 12 13 included the intention to treat analysis. Three studies 14 15 16 presented uncertain risk for randomization. For allocation bias, Daraï et al. 11 had an uncertain risk and Roman et al. 12 presented a low risk, while the others had a high risk. One study 15 reported high risk of bias and two were categorized as low-risk. 11 12 Regarding the rest of risk of bias, only Roman et al. 12 reported low risk of bias, whereas the others were labeled uncertain ( Fig. 2 ).

About the quality of evidence and the strength of recommendation according to the GRADE criteria, the studies presented a very low quality of evidence regarding reducing dyspareunia (MD: 1.18; 95%CI: 1.46–1.90; 3 studies, 167 women) and gastrointestinal complaints (KESS: MD: 1.63 for constipation; 95%CI: 1.82–1.43; 3 studies, 355 women; WEXNER: MD 0.25 for FI; 95%CI: 0.38–0.11; 3 studies, 355 women; GIQLI: MD 26.56 of gastrointestinal quality of life; 95% CI: 25.74–27.38; 3 studies, 355 women).

### Results from Individual Studies

Chart 1 describes the general characteristics of the studies selected for the review.

**Chart 1:** General characteristics of the included studies

Author/ Year	Sample	Intervention	Follow-up	Assessment methods	Preoperative PFDs	FI	UI	Dyspareunia	POP
Daraï et al. (2010) 11	*n* =52 Laparoscopy ( *n* = 26) versus open surgery ( *n* = 26)	Laparoscopy versus open surgery	1 and 6 months, then every 6 months up to 3 years	1. **Pain** VAS	There is no report in the study	There is no report in the study	There is no report in the study	When the overall result was assessed, there was a reduction in pain in both groups. However, when analyzed separately, the reduction of the complaint was not significant	There is no report in the study
Roman et al. (2018) 12	*n* = 60 Conservative surgery ( *n* = 27) versus Radical surgery ( *n* = 33)	Conservative surgery versus radical rectal resection	6-month intervals for 2 years	1. **Fecal Incontinence** GIQLI, Kess and Wexner score 2. **Urinary Incontinence** USP score 3. **Pain and quality of life** VAS and SF-36	Patients presented gastrointestinal disorders such as fecal incontinence and gas loss, in addition to dyspareunia in both groups studied	Both groups had gastrointestinal disorders after the 24-month evaluation, with no significant difference for the types of surgery	There is no report in the study	Despite the reduction in VAS in the groups studied, there was no significant difference for the types of surgery	There is no report in the study
Roman et al. (2019) 13	*n* = 55 Excision ( *n* = 27) versus Resection ( *n* = 28)	Excision versus Colorectal segmental resection	5 years	1. **Fecal Incontinence** GIQLI, Kess, and Wexner score 2. **Urinary Incontinence** USP score 3. **Pain and quality of life** VAS and SF-36	Patients presented gastrointestinal disorders such as fecal incontinence and gas loss, in addition to dyspareunia in both groups studied	Both groups had gastrointestinal disorders after a 5-year assessment. Despite the improvement when compared with preoperative values, there was no significant difference between the groups	There is no report in the study	Despite the reduction in VAS in the groups, there was no significant difference for the types of surgery	There is no report in the study
Roman et al. (2019) 14	*n* = 60 Conservative surgery ( *n* = 27) versus segmental resection ( *n* = 33)	Conservative surgery versus segmental resection	6,12,18 and 24 months	1. **Fecal Incontinence** GIQLI, Kess and Wexner score 2. **Urinary Incontinence** USP score	Patients had gastrointestinal disorders such as fecal incontinence and gas loss in both groups. The groups were not analyzed separately regarding the type of surgery	Both groups showed significant improvement after an evaluation when compared together. When analyzed separately, there was no significant difference between groups	There is no report in the study	There is no report in the study	There is no report in the study
Posadzka et al. (2015) 15	*n* = 48 Electroablation ( *n* = 33) versus CO2 laser ablation ( *n* = 15)	Electroablation of endometriosis versus CO2 laser ablation	3 and 6 months	1. **Pain** NRS	Both groups had dyspareunia.	There is no report in the study	There is no report in the study	After an initial improvement verified in 3 months, the complaint worsened significantly in the exam of 6 months for the CO2 laser group. For the electroablation group, the complaint also increased significantly, 50% of the patients reported a level ≥ 10 points after 6 months	There is no report in the study
Candiani et al. (1992) 16	*n* = 71 Conservative surgery ( *n* = 36) versus Presacral neurectomy ( *n* = 35)	Conservative surgery versus Presacral neurectomy	12 months	1. **Pain** Andersch and Milsom Multidimensional Scoring System	Both groups had dyspareunia (mild, moderate, and severe)	There is no report in the study	There is no report in the study	Although not significant, there was a reduction in complaints in both groups	There is no report in the study

Abbreviations: GIQLI, Gastrointestinal Quality of Life index; KESS, Knowles Eccersley Scott Symptom; NRS, Numerical Rating Scale; SF-36, Short Form Health Survey 36; USP, Urinary Symptom Profile; VAS, Visual Analogue Scale.

### Dyspareunia

Five studies 11 12 13 15 16 evaluated dyspareunia, with only one 16 specifying having assessed dyspareunia in depth. Daraï et al., 11 comparing laparoscopically assisted or open colorectal resection surgery techniques, found a significant improvement in dyspareunia after surgery, with a median pain of 1 (0 to 8) ( *p* < 0.0001), but with no difference between the techniques.

Roman et al. 12 evaluated the results after conservative surgery (shaving or disc excision) and radical rectal surgery (segmental resection), with no difference between groups after 24 months (median dyspareunia of 3 (2 to 3) and 4 (3 to 6), respectively; *p* = 1.00). In another study by the same group, 13 comparing shaving, disc excision or segmental resection 5 years after the surgery, they demonstrated a reduction in dyspareunia, with no statistically significant difference between the surgical techniques.

Posadzka et al. 15 compared electroablation versus laparoscopy CO2 laser ablation and found an improvement in dyspareunia at 3 months after surgery; however, at 6 months, there was an increase in the symptom score within both groups.

Candiani et al. 16 compared the surgical techniques of conservative surgery alone or conservative surgery with presacral neurectomy. The authors have found a reduction in moderate and severe dyspareunia and an increase in the number of asymptomatic women in both groups. However, they have concluded that presacral neurectomy did not add significant improvement in the performance of conservative surgery alone.

### Gastrointestinal Symptoms

Only three studies 12 13 14 evaluated the complaint of FI, classifying it as an involuntary loss of gas or feces, and they are from the same group. Roman et al. 12 compared shaving/disc excision versus segmental resection and, after 24 months, they found an improvement in FI symptoms within both groups, but with no difference between them ( *p* = 0.83). They also used assessment of gastrointestinal symptoms using the GIQLI score (low scores are related to a worse result) and, after treatment, the scores increased in both groups, but with no significant difference between them ( *p* = 0.64). Wexner scores before and after treatment behaved the same way ( *p* = 0.42).

The second study 13 presented a longer follow-up period (5 years) and the authors have also noticed symptom improvement, but with no difference between groups ( *p* = 0.42). The presurgical evaluation using the GIQLI, KESS, and Wexner score questionnaires showed improvement in the functional results, but with no difference between the groups. A third study 14 has found the same results.

### Subgroup Meta-analysis

**Fig. 3:**
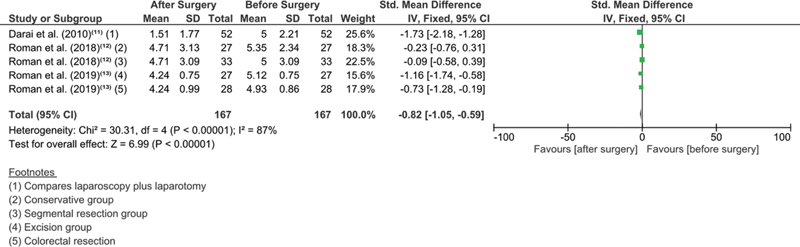
Subgroup analysis for dyspareunia comprising two studies across each group before and after surgery.

**Fig. 4:**
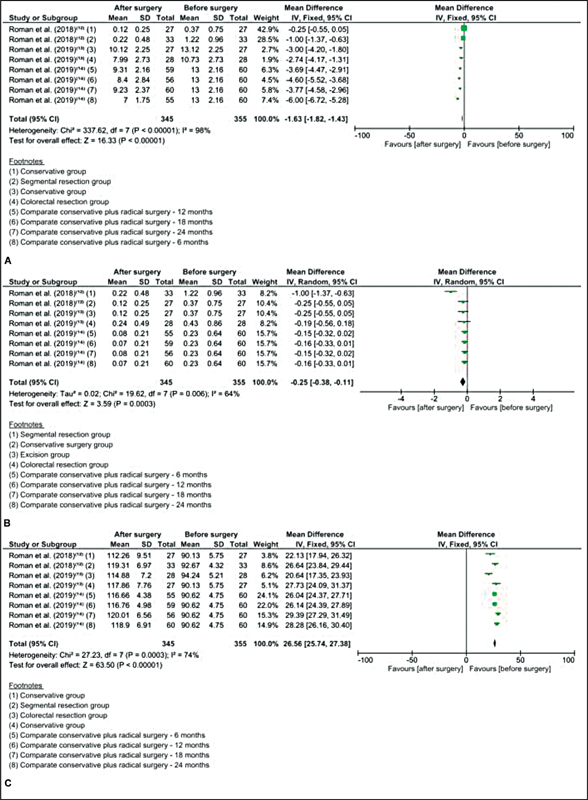
Subgroup analysis for gastrointestinal symptoms. ( **A** ) Subgroup analysis for gastrointestinal symptoms (KESS questionnaire) comprising two studies across each group before and after surgery. ( **B** ) Subgroup analysis for gastrointestinal symptoms (WEXNER questionnaire) comprising two studies across each group before and after surgery. ( **C** ) Subgroup analysis for gastrointestinal symptoms (GIQLI questionnaire) comprising two studies across each group before and after surgery.

In the subgroup meta-analysis, it can observed that there was a decrease in dyspareunia after surgical intervention (MD: - 0.82 [- 1.05–- 0.59] ( *p* < 0.00001) ( Fig. 3 ); however, an important heterogeneity is found as each study represents a different intervention (Chi 2 : 30.31; I ^2^ : 87%). The same can be observed for constipation ( Fig. 4 ) (assessed by the Kess questionnaire) ( Fig. 4a ) and FI (assessed by the Wexner scale) ( Fig. 4b ); there was an improvement for both (MD: - 1.63 [- 1.82–- 1.43]; *p* < 0.00001; MD: - 0.25 [- 0.38–- 0.11]; *p* = 0.006), with high heterogeneity (I ^2^ : 98 and 64%). We can observe the same pattern for gastrointestinal quality of life, with the improvement of the GIQLI questionnaire (MD: 26.56 [25.74–27.28]; *p* = 0.0003), with high heterogeneity (I ^2^ : 74%) ( Fig. 4c ).

## Discussion

Although the present review found studies that addressed the effect of the surgical treatment of DIE on pelvic floor dysfunctions, the heterogeneity of the studies did not make it possible to gather and analyze all the data. Within the subgroup analysis, it was possible to observe the benefits of surgical treatment for some pelvic floor disorders (dyspareunia and FI), but without superiority for a technique. According to the GRADE tool, the quality of the evidence was very low for both symptoms evaluated, that is, reduction of dyspareunia and improvement of gastrointestinal symptoms. None of the selected studies evaluated the presence and/or alteration of UI and POP.

Among the studies that analyzed dyspareunia, although most of them suggested a reduction in this symptom after surgical treatment, one of them 15 revealed a resurgence of the symptom at the same level after 6 months, indicating the need for long-term evaluations. Through the meta-analysis, it was possible to confirm the results presented individually by the authors; however, the high heterogeneity among them is noteworthy. In the same direction, a recent systematic review that included only two surgical techniques (laparoscopic excision compared with laparoscopic ablation) for endometriosis and their effects on dyspareunia showed that both reduced the symptom, with no difference between the two techniques. 17

Likewise, we can point out that studies are scarce in the analysis of the dyspareunia response; they are even more restricted to gastrointestinal symptoms, such as FI. Although we have demonstrated, through meta-analysis, the improvement of symptoms of FI, the evidence is also not robust enough to indicate the superiority of one technique over another, with important heterogeneity between studies. Considering noncomparative studies, Erdem et al., 18 in a cohort study of 48 women with DIE, assessed long-term functional results (postoperative bowel movement and FI) after rectal resection, showing improvement in FI. A cohort study by Riiskjaer et al. 19 that evaluated 128 patients, before and after laparoscopic intestinal resection, also observed an improvement in the evacuation procedure 1 year after surgery.

Gastrointestinal symptoms usually present before surgical intervention, according to some authors, can predict postoperative results, which are worse the greater the severity of symptoms, indicating that surgical removal of the lesions may not completely reduce the symptoms. 20 21 Such data indicate that symptoms related to the pelvic floor should be evaluated before the surgical procedure. Their presence can directly interfere with functional results after surgery, requiring long-term follow-up.

We did not find data regarding dysfunctions related to UI and POP that could be included in a robust methodological analysis, although the literature draws attention to the risk of impaired urinary control when DIE is surgically treated. 22 Considering the extent of endometriotic lesions and the extent of surgical procedures performed, a potential effect on such pelvic floor dysfunctions may occur.

Considering that one of the most important indications for the surgical treatment of DIE is the control of pain symptoms, the present review has its main strength in demonstrating that surgeries, regardless of the technique used, can reduce dyspareunia and intestinal complaints, but also it has its greatest weaknesses when it demonstrates the great heterogeneity between the studies about the comparator group and the different instruments used to evaluate the results, as well as differences between the follow-up period across studies. Thus, groups of experts must meet and indicate methodologies that guide the authors when planning and executing prospective controlled studies to treat symptomatic women with DIE, evaluating the possible implications on pelvic floor dysfunctions.

## Conclusion

Dyspareunia and FI improved after the surgical procedure, but it was not possible to demonstrate which surgical technique was related to these outcomes, as there was surgical heterogeneity. This diversity was found in the data, recommending future prospective studies addressing UI, POP and FI so that more robust evidence can be provided to health professionals about the association of DIE and pelvic floor disorders.
